# Comparison of Efficacy and Safety of First-Line Treatment Options for Unresectable Stage III Non-Small Cell Lung Cancer: A Retrospective Analysis

**DOI:** 10.1155/2024/8585035

**Published:** 2024-02-12

**Authors:** Luqing Zhao, Zhiting Zhao, Xiaoqi Yan, Fei Wu, Ning Sun, Renhong Guo, Shaorong Yu, Xiao Hu, Jifeng Feng

**Affiliations:** ^1^Department of Medical Oncology, The Affiliated Cancer Hospital of Nanjing Medical University & Jiangsu Cancer Hospital & Jiangsu Institute of Cancer Research, Nanjing, Jiangsu, China; ^2^Department of Oncology, The Air Force Hospital from Eastern Theater of PLA, Nanjing, Jiangsu, China; ^3^Department of Oncology, The Affiliated Suqian First People's Hospital of Nanjing Medical University & Suqian First Hospital, Suqian, Jiangsu, China

## Abstract

**Background:**

Based on PACIFIC trial, durvalumab as consolidation therapy following concurrent chemoradiotherapy (cCRT) has been a new standard treatment for unresectable stage III non-small cell lung cancer (NSCLC). In clinical applications, there are heterogeneous adjustments or novel strategies following specialized discussions in experienced multidisciplinary teams. This study retrospectively compared the efficacy and safety of different first-line treatments for unresectable stage III NSCLC.

**Methods:**

We retrospectively analyzed 397 patients who received first-line treatment for unresectable stage III NSCLC. Comparisons and statistical analyses of treatment were made in terms of efficacy and safety. Adverse events and responses were assessed using CTCAE v5.0 and RECIST v1.1. The progression-free survival (PFS) was estimated using the Kaplan–Meier method or the Cox survival regression model and compared using the log-rank test.

**Results:**

In wild-type driver genes group, the objective response rate (ORR), disease control rate (DCR), and median PFS (mPFS) were prolonged in the radiotherapy group compared to those in the nonradiotherapy group (ORR: 50.94% vs. 30.06%, *p* < 0.001; DCR: 98.11% vs. 80.37%, *p* < 0.001; and mPFS: 21.00 vs. 8.20 months, *p* < 0.001). The incidence of pneumonia at any grade in the radiotherapy group was higher than that in the nonradiotherapy group (9.43% vs. 2.45%, *p* = 0.008). In the radiotherapy group, the chemoradiotherapy (CRT) plus immunotherapy subgroup had longer mPFS than the CRT subgroup, with increased toxicity at any grade (24.60 vs. 17.90 months, p = 0.025, and 83.17% vs. 65.52%, *p* = 0.011). In the nonradiotherapy group, the DCR and mPFS were higher in the chemotherapy plus immunotherapy subgroup than in the chemotherapy subgroup, with increased toxicity at any grade (DCR: 93.67% vs. 67.86%, *p* < 0.001; mPFS: 13.53 vs. 5.07 months, *p* < 0.001; and 68.35% vs. 41.67%, *p* = 0.001). In the mutant driver genes group, the efficacy did not significantly differ among the radiotherapy subgroup, targeted therapy subgroup, and radiotherapy plus targeted therapy subgroup (ORR: *p* = 0.633; mPFS: *p* = 0.450).

**Conclusions:**

For unresectable stage III NSCLC patients with wild-type driver genes, the combination of radiotherapy and immunotherapy in the initial treatment was essential to significantly improve the efficacy. For patients with mutant driver genes, radiotherapy, targeted therapy, and the combination of radiotherapy and targeted therapy showed similar short-term efficacy.

## 1. Introduction

Lung cancer is the second most commonly diagnosed cancer and the leading cause of cancer death, with an estimated 2.2 million new cases and 1.8 million deaths in 2020 [1]. Non-small cell lung cancer (NSCLC) accounts for about 85% of all the diagnosed lung cancer cases and stage III disease accounts for approximately one third of these cases. The expected 5-year survival rate for patients with unresectable stage III NSCLC is between 13% and 36% [2].

The treatment goal for patients with unresectable stage III NSCLC is to prevent local recurrence and reduce the occurrence of distant metastases. Concurrent chemoradiotherapy (cCRT) is the traditional standard therapy for this population, which has reported positive results in several phase III clinical trials and meta-analyses. In the RTOG 9410 trial, cCRT showed a long-term survival benefit compared with sequential chemoradiotherapy (sCRT) (median overall survival: 17.0 vs. 14.6 months; 5-year survival rate: 16% vs. 10%) [3]. Unfortunately, most patients progress after cCRT, with median progression-free survival (mPFS) of 8–12 months and a 5-year overall survival (OS) rate of 15–25% [4]. Subsequent phase II/III trials on the cCRT-based integrated approach (CALGB III, RTOG0617 phase II, and SWOGS0023 phase III) similarly failed to show improved survival in such patients [5–7].

In 2017, the PACIFIC trial, a phase III placebo-controlled trial, demonstrated that patients with unresectable stage III NSCLC who were treated with durvalumab, as consolidation therapy following cCRT, experienced significantly better survival outcomes compared to placebo (PFS: 16.8 vs. 5.6 months; HR = 0.52; 95% CI, 0.42–0.65, *p* < 0.001; 5-year rates for OS: 42.9% vs. 33.4%; and 5-year rates for PFS: 33.1% vs. 19.0%) [8, 9]. Based on these encouraging results, programmed death ligand-1 (PD-L1) inhibitors durvalumab as consolidation therapy following cCRT has been a new standard treatment for unresectable stage III NSCLC. Since then, some further studies (LUN14-179 trial and DETERRED trial) have confirmed that other checkpoint inhibitors as consolidation therapy after cCRT are similar to the efficacy of durvalumab, supporting the importance of immunotherapy in this new era of treatment [10, 11].

Stage III NSCLC comprises a heterogeneous group of patients, for whom dedicated discussion within an experienced multidisciplinary team is mandatory. A retrospective series showed that only half of the patients with stage III NSCLC are treated with CRT in clinical practice [12]. Besides, not all the patients treated with cCRT are eligible for adjuvant durvalumab due to residual toxicity and disease progression [13, 14]. The update results from the PACIFIC trial showed that PFS and OS in the epidermal growth factor receptor (EGFR) mutation-positive group were significantly lower than those in the whole group and the EGFR mutation-negative group [9, 15]. Also, in multiple immunotherapy-related clinical trials for advanced lung cancer, results have shown that patients with EGFR mutation do not benefit from immunotherapy [16–18]. According to these, in some clinical applications, targeted monotherapy or combination therapy is used to treat unresectable stage III NSCLC patients carrying driver gene mutations. An exploratory analysis from the pacific study also suggests that durvalumab treatment given early (≤14 days) after cCRT may benefit more [8]. Therefore, the simultaneous combination of programmed cell death protein-1 (PD-1)/PD-L1 inhibitors with cCRT has potential clinical benefits. As of now, several larger phase III trials of immunosynchronous therapy modalities are ongoing, including NCT03519971 and NCT04092283. In some clinical applications, the time of immunotherapy combined with CRT is advanced, that is, from immunotherapy consolidation therapy to immunotherapy synchronization therapy. The aim of this study was to analyze and compare the efficacy and safety of different first-line treatment options for unresectable stage III NSCLC.

## 2. Methods

### 2.1. Patients

We retrospectively reviewed the medical records of 397 patients with unresectable stage III NSCLC from January 1, 2013, to April 30, 2023. Study measures (i.e., patients' basic information, clinical characteristics, and treatment patterns) were summarized with descriptive statistics. The inclusion criteria were as follows: (1) patients histologically or cytologically diagnosed with unresectable stage III NSCLC; (2) patients who scored 0–2 on the Eastern Cooperative Oncology Group performance status (ECOG PS); (3) patients who had not received any previous treatment; and (4) patients included in this study had at least one measurable disease. The study was conducted in accordance with the Declaration of Helsinki (as revised in 2013). The study was approved by the Academic Ethics Committee of Jiangsu Cancer Hospital, approval number (no. [2018]074), and individual consent for this retrospective analysis was waived.

### 2.2. Data Collection and Response Assessment

Medical records were reviewed and extracted on clinical pathologic features and treatment histories. Treatment outcomes were the extracted objective response rate (ORR) and the disease control rate (DCR), along with PFS after the start of first-line therapy. The ORR was defined as the complete response (CR) or partial response (PR) rate. The DCR was defined as the percentage of patients with the ORR and stable disease (SD). The PFS was defined as the time between the date of treatment initiation and the date of progressive, death, or last follow-up. Response was assessed using the Response Evaluation Criteria in Solid Tumors (RECIST) version 1.1. Adverse events (AEs) were graded according to the Common Terminology Criteria for Adverse Events (CTCAE) version 5.0.

### 2.3. Statistical Analysis

Categorical variables were compared using the *χ*2 test or Fisher's exact test. The PFS was determined by the Kaplan–Meier method and compared by the log-rank test. The Cox proportional model was used to evaluate the various prognostic factors. All statistical analyses were conducted using the SPSS (version 26.0) and R software (version 3.6.3). *p* < 0.05 was considered statistically significant.

## 3. Result

### 3.1. Patient Characteristics

A total of 397 patients with unresectable stage III NSCLC received first-line treatment from January 1, 2013, to April 30, 2023. The patients were divided into two groups. There were 322 patients in the wild-type driver genes group and 75 patients in the mutant driver genes group. Baseline clinical and pathological features are summarized in Table 1.

### 3.2. Treatment Characteristics

All 397 patients were assessable for response (Table 1). In the wild-type driver genes group, 159 (49.38%) patients received radiotherapy and 163 (50.62%) patients did not receive radiotherapy. In the radiotherapy group, 58 patients received CRT with or without bevacizumab and 101 patients were treated with CRT plus immunotherapy. In the nonradiotherapy group, 84 patients received chemotherapy with or without bevacizumab and 79 patients received chemotherapy plus immunotherapy. In the mutant driver genes group, 14 patients received radiotherapy with or without chemotherapy, 31 were treated with targeted therapy with or without chemotherapy, and 16 received radiotherapy plus targeted therapy with or without chemotherapy.

### 3.3. Efficacy

**Figure 1:**
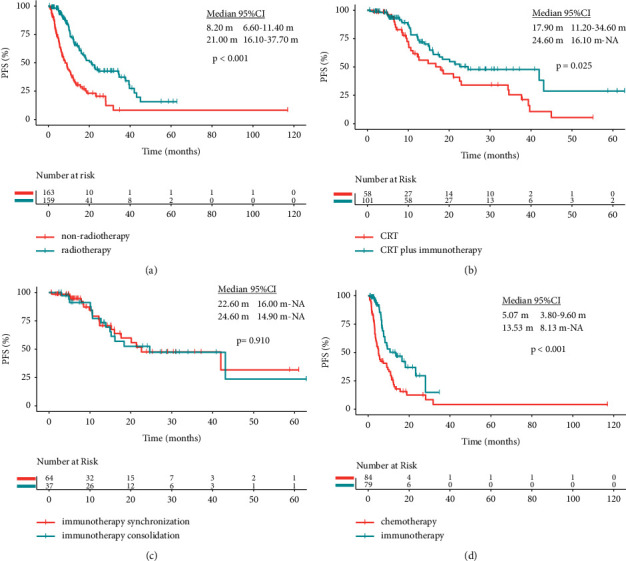
Kaplan–Meier curves in the wild-type driver genes group. (a) Progression-free survival (PFS) in the non-radiotherapy group and radiotherapy group. (b) PFS in the CRT group and the CRT plus immunotherapy group. (c) PFS comparison between immunotherapy synchronization therapy and immunotherapy consolidation therapy in the CRT plus immunotherapy group. (d) PFS in the chemotherapy group and the immunotherapy plus chemotherapy group for patients with non-radiotherapy.

In the wild-type driver genes group, the ORR, DCR, and mPFS were prolonged in the radiotherapy group than those in the nonradiotherapy group (ORR: 50.94% vs. 30.06%, *p* < 0.001; DCR: 98.11% vs. 80.37%, *p* < 0.001; and mPFS: 21.00 vs. 8.20 months, *p* < 0.001, Figure 1(a)). Furthermore, in the radiotherapy group, the CRT plus immunotherapy subgroup had longer mPFS than the CRT subgroup (24.60 vs. 17.90 months, *p* = 0.025, Figure 1(b)). The ORR of the immunotherapy consolidation therapy subgroup was higher than that of the immunotherapy synchronization therapy subgroup (67.57% vs. 42.19%, *p* = 0.014, Table 2). But there was no statistical difference in mPFS between the two groups (24.60 vs. 22.60 months, *p* = 0.910, Figure 1(c)). In the nonradiotherapy group, the DCR and mPFS of the chemotherapy plus immunotherapy subgroup were significantly higher than those of the chemotherapy subgroup (DCR: 93.67% vs. 67.86%, *p* < 0.001 and mPFS: 13.53 vs. 5.07 months, *p* < 0.001, Figure 1(d)).

**Figure 2:**
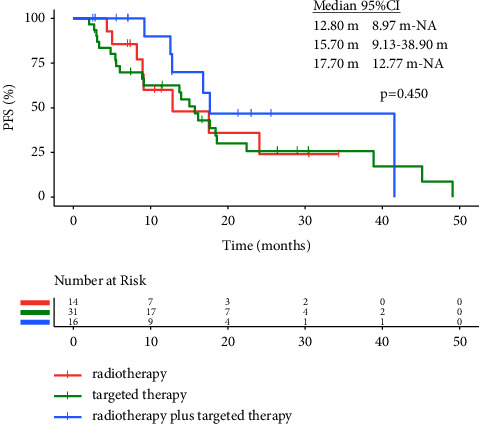
Kaplan–Meier curves in the mutant driver genes group. PFS in the radiotherapy group, targeted therapy group, and radiotherapy plus targeted therapy group.

In the mutant driver genes group, the efficacy did not significantly differ among the radiotherapy subgroup, targeted therapy subgroup, and radiotherapy plus targeted therapy subgroup (ORR: 57.14% vs. 48.39% vs. 62.50%, *p* = 0.633, and mPFS: 12.80 vs. 15.70 vs. 17.70 months, *p* = 0.450, Figure 2).

With or without radiotherapy was a significant factor affecting the PFS in the wild-type driver genes group (*p* < 0.001, Table 3). Age, sex, history of smoke, ECOG score, and histology were not the major factors affecting the PFS in both groups.

### 3.4. Safety

As shown in Table 4, in the wild-type driver genes group, 65.53% (211/322) of the patients experienced treatment-related AEs and 19.88% (64/322) patients experienced grade 3 or 4 treatment-related AEs. The most common treatment-related AEs was anemia (23.29%), followed by leukopenia (21.43%), neutropenia (21.43%), thrombocytopenia (14.60%), and elevated alanine aminotransferase (ALT) or aspartate transaminase (AST) (9.63%). No grade 5 treatment-related AEs was reported. Compared to the nonradiotherapy group, the incidence of AEs at any grade was higher in the radiotherapy group (76.73% vs. 54.60%, *p* < 0.001). There was no significant difference in the incidence of AEs at grade 3 and grade 4 between the two groups (22.64% vs. 17.28%, *p* = 0.219). The incidence of pneumonia at any grade in the radiotherapy group was higher than that in the nonradiotherapy group (9.43% vs. 2.45%, *p* = 0.008), among which radiation-related pneumonia accounted for 73.33%. But the difference in the incidence of pneumonia at grade 3 and grade 4 between the two groups was not statistically significant (1.89% vs. 0.61%, *p* = 0.367). In the nonradiotherapy group, the incidence of AEs at any grade in the chemotherapy plus immunotherapy subgroup was significantly higher compared to that in the chemotherapy subgroup (68.35% vs. 41.67%, *p* = 0.001) and there was no significant difference in the incidence of AEs at grade 3 and grade 4 between the two groups (20.25% vs. 14.29%, *p* = 0.313). In the radiotherapy group, the incidence of AEs at any grade in the CRT plus immunotherapy group was significantly higher than that in the chemotherapy group (83.17% vs. 65.52%, *p* = 0.011); however, grade 3 or 4 toxicity was similar in both groups (23.76% vs. 20.69%, *p* = 0.656).

As shown in Table 5, among the treatment-related AEs in the mutant driver genes group, neutropenia had the highest incidence (22.67%), followed by anemia (20.00%), leukopenia (17.33%), elevated ALT or AST (13.33%), and febrile neutropenia (10.67%). No statistically significant differences in the incidence of AEs were observed among the radiotherapy subgroup, targeted therapy subgroup, and radiotherapy plus targeted therapy subgroup (any grade: 64.29% vs. 29.03% vs. 31.25%, *p* = 0.064 and grade 3/4 : 21.43% vs. 9.68% vs. 12.50%, *p* = 0.541).

## 4. Discussion

This study retrospectively compared the efficacy and safety of different first-line treatments for unresectable stage III NSCLC after grouping patients according to wild type or mutant driver genes. In the wild-type driver genes group, the presence of radiotherapy significantly improved efficacy. Single-modality radiotherapy was the standard for unresectable stage III NSCLC in the 1980s based on the RTOG 7301 trial [19]. In addition, some following studies demonstrated improvement in symptoms after radiation treatment [20]. Our results are consistent with these results, which suggest that radiotherapy is associated with improved survival in patients with unresected stage III NSCLC. And, further multivariate analysis showed that radiotherapy was indeed a significant factor affecting the PFS in the wild-type driver genes group (*p* < 0.001).

In order to meet the urgent need for better efficacy, there has been progress in the treatment modalities for unresectable stage III NSCLC. Recently, immunotherapy has shown striking survival improvement in unresectable stage III NSCLC. Our study showed similar positive findings. Further subgroup analysis showed that immunotherapy had significantly better survival compared to chemotherapy, whether patients are with or without radiotherapy in the wild-type driver genes group. Also, the most obvious improvement was found in the immunotherapy in combination with the CRT group, which further confirms the results of the PACIFIC trial. The potential reason might be that the drugs of PD-1/PD-L1 checkpoint inhibition could synergize the effect of CRT. Since radiotherapy generates in situ vaccination which can be substantially potentiated by immunotherapy, the abscopal effect of radiotherapy has become more meaningful [21]. Furthermore, radiotherapy can stimulate antitumor adaptive immunity, modulating the tumor microenvironment and inducing tumor PD-L1 levels [21–23].

Collectively, for patients with wild-type driver genes, the combination of radiotherapy and immunotherapy is a critical component of definitive treatment to significantly improve outcomes. At present, there are some clinical trials to explore further progress based on the success of the combination of CRT and immunotherapy. An exploratory analysis of the PACIFIC study also suggests that durvalumab treatment given earlier (≤14 days) after cCRT may benefit more. Subgroup analysis found that the subgroup initiated with durvalumab ≤2 weeks after radiotherapy significantly delayed disease progression. This suggests the potential for simultaneous immunotherapy with CRT, but this model is still in the exploratory stage. Preliminary data from the phase II clinical study DETERRED showed that atezolizumab is feasible when administered concurrently with CRT followed by chemotherapy-atezolizumab consolidation. This treatment strategy did not increase the incidence of radiation-related pneumonia in terms of safety. In terms of effectiveness, the rates of 1-year PFS and OS are 66% and 77%, respectively. It appears to be better on the rate of 1-year PFS (55.9%) compared to the PACIFIC, but the rate of 1-year OS (83.1%) is slightly worse [11]. At the same time, the ETOPNICOLAS phase II clinical study also showed that cCRT synchronization with nivolumab and then nivolumab maintenance in unresectable stage III NSCLC was feasible. Also, no increased risk of unintended adverse events or severe pneumonia was observed. The median PFS was 12.7 months, the median OS was 38.8 months, and the 1-year PFS and OS rates were 53.7% and 75.7%, respectively [24]. In our study, the ORR in the synchronization therapy subgroup was lower than that in the consolidation therapy subgroup. However, this was a retrospective, nonrandomized study, which may have led to bias in the results due to the limited sample size. And, there was no statistical difference in mPFS and the rate of pneumonia between the two groups. Our study, like previous trials, suggests the potential for simultaneous immunotherapy with CRT. Several large-scale phase III trials of immunosynchronous therapy modalities are ongoing, including NCT03519971 and NCT04092283.

There is a concern that toxicity could be further aggravated when immunotherapy and radiotherapy are given. Although the radiotherapy group increased the rate of pneumonia at any grade (9.43%), there was no statistical difference in the incidence of grade 3/4 pneumonia between the two groups. Furthermore, in the PACIFIC trial, rates of grade 3/4 AEs of any cause were similar for durvalumab compared with placebo (29.9% vs. 26.1%) [25]. Several prospective trials (LUN14-179 and BTCRC LUN16-081) have also confirmed that the toxicity of consolidation immunotherapy after CRT is tolerable [10, 26]. Similarly, our study showed no differences in grade 3/4 AEs between the immunotherapy group and the CRT or chemotherapy group.

About patients with EGFR mutations, the subgroup analysis of the PACIFIC trial showed that they might not benefit from maintenance immunotherapy [15]. ESMO expert consensus does not recommend the use of adjunctive durvalumab in patients with EGFR mutations. Another treatment should thus be explored for this population. Some studies suggest many stage III NSCLC patients have driver mutations such as EGFR (10–30% of the patients) [27]. In our study, EGFR-mutant patients account for about 50% in the mutant driver genes group while others have anaplastic lymphoma kinase gene rearrangements (ALK+), v-raf murine sarcoma viral oncogene homolog B (BRAF) mutations, and so on. Among patients with advanced NSCLC with oncogenic driver mutations, molecular-targeted drugs have been recommended for the first-line therapy, which dramatically changed the standard treatment. Preclinical studies have shown that EGFR tyrosine kinase inhibitors (TKIs) could have a radio-sensitizing effect, which showed the combination of EGFR-TKIs and radiotherapy seems to be a reasonable approach [28, 29]. However, there are few recommendations indicating whether radiotherapy is effective for patients with mutant driver genes. Thus, it was worth to explore the efficacy of radiotherapy for this population given that it significantly improved the survival for patients with wild-type driver genes. In our study, there were no differences in short-term efficacy among the radiotherapy subgroup, targeted therapy subgroup, and radiotherapy plus targeted therapy group, which showed radiotherapy seemly failed to improve outcomes whether combined with targeted therapy. Although several phase II clinical experiments (RECEL trial and WJOG6911L trial) showed the potential value of concurrent targeted therapy with radiotherapy [30, 31], no phase III trials have yet demonstrated similar results. Based on the abovementioned results, it may not be urgent to add radiotherapy to first-line treatment to improve the efficacy for patients with mutant gene driver. However, the limited number of patients and the retrospective nature of this analysis do not lead to obtain firm conclusions, and further prospective studies should aim to confirm these results.

There are several limitations in this study. This analysis was based on a small sample from a single institution. Also, the study was retrospective in design, which is inherently affected by selection bias and missing data. Moreover, reliance on electronic health records may mean that some events may be underestimated. Therefore, our results suggest the possibility of clinical treatment rather than reaching definitive conclusions. Larger prospective studies are needed to confirm our findings.

## 5. Conclusions

For unresectable stage III NSCLC patients with wild-type driver genes, the combination of radiotherapy and immunotherapy in the initial treatment was essential to significantly improve the efficacy. For patients with mutant driver genes, radiotherapy, targeted therapy, and the combination of radiotherapy and targeted therapy showed similar short-term efficacy.

## Figures and Tables

**Table 1:** Clinical characteristics of patients and clinical activity of first-line treatment.

Characteristics	Wild-type driver genes group (*n* = 322)									Mutant driver genes group (*n* = 75)								
	Patients number (%)	CR	PR	SD	PD	ORR (%)	*p*	DCR (%)	*p*	Patients number (%)	CR	PR	SD	PD	ORR (%)	*p*	DCR (%)	*p*
Sex																		
Female	36 (11.18)	0	16	18	2	44.44	0.597	94.44	0.422	23 (30.67)	0	12	10	1	52.17	0.743	95.65	1.000
Male	286 (88.82)	0	114	139	33	39.86		88.46		52 (69.33)	0	25	24	3	48.08		94.23	
Age																		
≤60	70 (21.74)	0	33	27	10	47.14	0.192	85.71	0.299	28 (37.33)	0	10	15	3	35.71	0.069	89.29	0.144
>60	252 (78.26)	0	97	130	25	38.49		90.08		47 (62.67)	0	27	19	1	57.45		97.87	
History of smoking																		
No	249 (77.33)	0	97	124	28	38.96	0.339	88.76	0.689	64 (85.33)	0	31	30	3	48.44	0.708	95.31	0.477
Yes	73 (22.67)	0	33	33	7	45.21		90.41		11 (14.67)	0	6	4	1	54.55		90.91	
ECOG performance status																		
0	53 (16.46)	0	20	25	8	37.74	0.840	84.91	0.486	18 (24.00)	0	8	9	1	44.44	0.670	94.44	1.000
1	255 (79.19)	0	105	125	25	41.18		90.20		47 (62.67)	0	25	19	3	53.19		93.62	
2	14 (4.35)	0	5	7	2	35.71		85.71		10 (13.33)	0	4	6	0	40.00		100.0	
Histology																		
Adenocarcinoma	104 (32.30)	0	36	58	10	34.62	0.300	90.38	0.764	65 (86.67)	0	34	28	3	52.31	0.285	95.38	0.191
Squamous	204 (63.35)	0	87	93	24	42.65		88.24		3 (4.00)	0	0	2	1	0		66.67	
Other	14 (4.35)	0	7	6	1	50.00		92.86		7 (9.33)	0	3	4	0	42.86		100.00	
Combined with radiotherapy																		
No	163 (50.62)	0	49	82	32	30.06	<0.001^*∗*^	80.37	<0.001^*∗*^	31 (41.33)	0	19	12	0	61.29	0.082	100.00	0.138
Yes	159 (49.38)	0	81	75	3	50.94		98.11		44 (58.67)	0	18	22	4	40.91		90.91	
Treatment with radiotherapy																		
Chemotherapy	58 (18.01)	0	29	28	1	50.00	0.857	98.28	1.000	—	—	—	—	—	—	—	—	—
Chemotherapy plus immunotherapy	101 (31.37)	0	52	47	2	51.49		98.02		—	—	—	—	—	—		—	
Treatment without radiotherapy										—								
Chemotherapy	84 (26.09)	0	21	36	27	25.00	0.146	67.86	<0.001^*∗*^	—	—	—	—	—	—	—	—	—
Chemotherapy plus immunotherapy	79 (24.53)	0	28	46	5	35.44		93.67		—	—	—	—	—	—		—	
Treatment in mutant driver genes																		
Radiotherapy	—	—	—	—	—	—	—	—	—	14 (18.67)	0	8	6	0	57.14	0.633	100	0.729
Targeted therapy	—	—	—	—	—	—	—	—	—	31 (41.33)	0	15	14	2	48.39		93.55	
Radiotherapy plus targeted therapy	—	—	—	—	—	—	—	—	—	16 (21.33)	0	10	6	0	62.50		100	
Total	322	0	130	157	35	40.37		89.13		75	0	37	34	4	49.33		94.67	

CR, complete response; PR, partial response; SD, stable disease; PD, progressive disease; ORR, objective response rate; DCR, disease control rate; ^*∗*^*p* < 0.05; —, not applicable.

**Table 2:** The efficacy of different subgroups in the CRT plus immunotherapy group.

	Immunotherapy consolidation therapy (*n* = 37)	Immunotherapy synchronization therapy (*n* = 64)	*p*
CR	0	0	
PR	25	27	
SD	11	36	
PD	1	1	
ORR (%)	67.57	42.19	0.014
DCR (%)	97.30	98.44	1.000
Any grade toxicities (%)	83.78	82.81	0.900
Pneumonia (%)	8.11	14.06	0.567
Grade 3-4 toxicities (%)	18.92	26.56	0.385
Pneumonia (%)	2.70	3.13	1.000

**Table 3:** Univariate analysis of factors of progression-free survival.

	Wild-type driver genes group		Mutant driver genes group	
	HR (95% CI)	*p* value	HR (95% CI)	*p* value
Age	1.10 (0.75–1.60)	0.600	1.40 (0.72–2.70)	0.320
Sex	1.50 (0.86–2.70)	0.150	1.00 (0.56–1.90)	0.920
Smoke	0.78 (0.52–1.20)	0.230	1.60 (0.65–3.80)	0.310
ECOG score 0 vs. ECOG score 1 vs. ECOG score 2	1.00 (0.67–1.60)	0.890	1.10 (0.60–2.00)	0.770
Histology	1.10 (0.83–1.50)	0.460	1.20 (0.72–1.90)	0.520
Radiotherapy vs. nonradiotherapy	0.36 (0.26–0.50)	<0.001^*∗*^	0.58 (0.31–1.10)	0.096

^*∗*^ *p* < 0.05.

**Table 4:** Treatment-related adverse events for total patients and patients with wild-type driver genes.

Events	Total patients (*n* = 322)		Nonradiotherapy (*n* = 163) vs. radiotherapy (*n* = 159)				Nonradiotherapy group				Radiotherapy group			
							Chemotherapy (*n* = 84) vs. chemotherapy plus immunotherapy (*n* = 79)				CRT (*n* = 58) vs. CRT plus immunotherapy (*n* = 101)			
	Any grade	Grade 3 or 4	Any grade	*p*	Grade 3 or 4	*p*	Any grade	*p*	Grade 3 or 4	*p*	Any grade	*p*	Grade 3 or 4	*p*
Any event	211 (65.53)	64 (19.88)	89 vs. 122 (54.60 vs. 76.73)	<0.001	28 vs. 36 (17.18 vs. 22.64)	0.219	35 vs. 54 (41.67 vs. 68.35)	0.001	12 vs. 16 (14.29 vs. 20.25)	0.313	38 vs. 84 (65.52 vs. 83.17)	0.011	12 vs. 24 (20.69 vs. 23.76)	0.656
Leukopenia	69 (21.43)	21 (6.52)	21 vs. 48 (12.88 vs. 30.19)		7 vs. 14 (4.29 vs. 8.81)		12 vs. 9 (14.29 vs. 11.39)		4 vs. 3 (4.76 vs. 3.80)		23 vs. 25 (39.66 vs. 24.75)		5 vs. 9 (8.62 vs. 8.91)	
Thrombocytopenia	47 (14.60)	8 (2.48)	13 vs. 34 (7.98 vs. 21.38)		1 vs. 7 (0.61 vs. 4.40)		4 vs. 9 (4.76 vs. 11.39)		1 vs. 0 (1.19 vs. 0)		14 vs. 20 (24.14 vs. 19.80)		5 vs. 2 (8.62 vs. 1.98)	
Neutropenia	69 (21.43)	24 (7.45)	25 vs. 44 (15.34 vs. 27.67)		13 vs. 11 (7.98 vs. 6.92)		9 vs. 16 (10.71 vs. 20.25)		3 vs. 10 (3.57 vs. 12.66)		15 vs. 29 (25.86 vs. 28.71)		4 vs. 7 (6.90 vs. 6.93)	
Anemia	75 (23.29)	5 (1.55)	29 vs. 46 (17.79 vs. 28.93)		5 vs. 0 (3.07 vs. 0)		9 vs. 20 (10.71 vs. 25.32)		2 vs. 3 (2.38 vs. 3.80)		11 vs. 35 (18.97 vs. 34.65)		0 vs. 0 (0 vs. 0)	
Febrile neutropenia	25 (7.76)	24 (7.45)	15 vs. 10 (9.20 vs. 6.29)		14 vs. 10 (8.59 vs. 6.29)		4 vs. 11 (4.76 vs. 13.92)		4 vs. 10 (4.76 vs. 12.66)		4 vs. 6 (6.90 vs. 5.94)		4 vs. 6 (6.90 vs. 5.94)	
Nausea/vomiting	11 (3.42)	1 (0.31)	4 vs. 7 (2.45 vs. 4.40)		0 vs. 1 (0 vs. 0.63)		4 vs. 0 (4.76 vs. 0)		0 vs. 0 (0 vs. 0)		3 vs. 4 (5.17 vs. 3.96)		0 vs. 1 (0 vs. 0.99)	
Decreased appetite	15 (4.66)	1 (0.31)	9 vs. 6 (5.52 vs. 3.77)		1 vs. 0 (0.61 vs. 0)		8 vs. 1 (9.52 vs. 1.27)		1 vs. 0 (1.19 vs. 0)		1 vs. 5 (1.72 vs. 4.95)		0 vs. 0 (0 vs. 0)	
Pneumonia	19 (5.90)	4 (1.24)	4 vs. 15 (2.45 vs. 9.43)	0.008	1 vs. 3 (0.61 vs. 1.89)	0.367	0 vs. 4 (0 vs. 5.06)		0 vs. 1 (0 vs. 1.27)		3 vs. 12 (5.17 vs. 11.88)		0 vs. 3 (0 vs. 2.97)	
Elevated ALT or AST	31 (9.63)	0	12 vs. 19 (7.36 vs. 11.95)		0 vs. 0 (0 vs. 0)		3 vs. 9 (3.57 vs. 11.39)		0 vs. 0 (0 vs. 0)		2 vs. 17 (3.45 vs. 16.83)		0 vs. 0 (0 vs. 0)	
Fatigue	7 (2.17)	0	6 vs. 1 (3.68 vs. 0.63)		0 vs. 0 (0 vs. 0)		3 vs. 3 (3.57 vs. 3.80)		0 vs. 0 (0 vs. 0)		0 vs. 1 (0 vs. 0.99)		0 vs. 0 (0 vs. 0)	
Hypothyroidism	3 (0.93)	0	1 vs. 2 (0.61 vs. 1.26)		0 vs. 0 (0 vs. 0)		0 vs. 1 (0 vs. 1.27)		0 vs. 0 (0 vs. 0)		0 vs. 2 (0 vs. 1.98)		0 vs. 0 (0 vs. 0)	
Rash	7 (2.17)	0	3 vs. 4 (1.84 vs. 2.52)		0 vs. 0 (0 vs. 0)		1 vs. 2 (1.19 vs. 2.53)		0 vs. 0 (0 vs. 0)		1 vs. 3 (1.72 vs. 2.97)		0 vs. 0 (0 vs. 0)	
Myocarditis	1 (0.31)	0	1 vs. 0 (0.61 vs. 0)		0 vs. 0 (0 vs. 0)		0 vs. 1 (0 vs. 1.27)		0 vs. 0 (0 vs. 0)		0 vs. 0 (0 vs. 0)		0 vs. 0 (0 vs. 0)	

**Table 5:** Treatment-related adverse events for patients with mutant driver genes.

Events	Total patients (*n* = 75)		Radiotherapy (*n* = 14) vs. targeted therapy (*n* = 31) vs. radiotherapy plus targeted therapy (*n* = 16)			
	Any grade	Grade 3 or 4	Any grade	*p*	Grade 3 or 4	*p*
Any event	37 (49.33)	14 (18.67)	9 vs. 9 vs. 5 (64.29 vs. 29.03 vs. 31.25)	0.064	3 vs. 3 vs. 2 (21.43 vs. 9.68 vs. 12.50)	0.541
Leukopenia	13 (17.33)	5 (6.67)	6 vs. 0 vs. 1 (42.86 vs. 0 vs. 6.25)		2 vs. 0 vs. 1 (14.29 vs. 0 vs. 6.25)	
Thrombocytopenia	7 (9.33)	2 (2.67)	2 vs. 1 vs. 1 (14.29 vs. 3.23 vs. 6.25)		1 vs. 0 vs. 0 (7.14 vs. 0 vs. 0)	
Neutropenia	17 (22.67)	4 (5.33)	6 vs. 2 vs. 2 (42.86 vs. 6.45 vs. 12.50)		0 vs. 2 vs. 2 (0 vs. 6.45 vs. 12.50)	
Anemia	15 (20.00)	1 (1.33)	4 vs. 2 vs. 1 (28.57 vs. 6.45 vs. 6.25)		0 vs. 0 vs. 0 (0 vs. 0 vs. 0)	
Febrile neutropenia	8 (10.67)	8 (10.67)	0 vs. 2 vs. 0 (0 vs. 6.45 vs. 0)		0 vs. 2 vs. 0 (0 vs. 6.45 vs. 0)	
Nausea/vomiting	5 (6.67)	0	2 vs. 2 vs. 0 (14.29 vs. 6.45 vs. 0)		0 vs. 0 vs. 0 (0 vs. 0 vs. 0)	
Decreased appetite	4 (5.33)	0	1 vs. 1 vs. 0 (7.14 vs. 3.23 vs. 0)		0 vs. 0 vs. 0 (0 vs. 0 vs. 0)	
Pneumonia	2 (2.67)	0	1 vs. 0 vs. 0 (7.14 vs. 0 vs. 0)		0 vs. 0 vs. 0 (0 vs. 0 vs. 0)	
Elevated ALT or AST	10 (13.33)	1 (1.33)	2 vs. 2 vs. 2 (14.29 vs. 6.45 vs. 12.50)		0 vs. 1 vs. 0 (0 vs. 3.23 vs. 0)	
Fatigue	1 (1.33)	0	0 vs. 0 vs. 0 (0 vs. 0 vs. 0)		0 vs. 0 vs. 0 (0 vs. 0 vs. 0)	
Hypothyroidism	2 (2.67)	0	0 vs. 0 vs. 1 (0 vs. 0 vs. 6.25)		0 vs. 0 vs. 0 (0 vs. 0 vs. 0)	
Rash	2 (2.67)	0	0 vs. 2 vs. 0 (0 vs. 6.45 vs. 0)		0 vs. 0 vs. 0 (0 vs. 0 vs. 0)	

Number of patients with an event (percent). ALT, alanine aminotransferase; AST, aspartate transaminase.

## Data Availability

The data used to support this study are available from the corresponding author upon request.

## Additional Points

*What is Already Known about This Topic?* Based on the PACIFIC trial, durvalumab as consolidation therapy following concurrent chemoradiotherapy (cCRT) has been a new standard treatment for unresectable stage III non-small cell lung cancer (NSCLC). *What Does This Article Add?* For patients with wild-type driver genes, the combination of radiotherapy and immunotherapy in the initial treatment was essential to significantly improve the efficacy. For patients with mutant driver genes, radiotherapy, targeted therapy, and the combination of radiotherapy and targeted therapy showed similar short-term efficacy.
